# Supplementary material for: TLR4 signalling via Piezo1 engages and enhances the macrophage mediated host response during bacterial infection
Source: Nat Commun. 2021 Jun 10;12:3519. doi: 10.1038/s41467-021-23683-y (PMC8192512; doi:10.1038/s41467-021-23683-y)
Supplement: Supplementary file 1 — Supplementary Information [file 41467_2021_23683_MOESM1_ESM.pdf]

Supplementary Figure 1

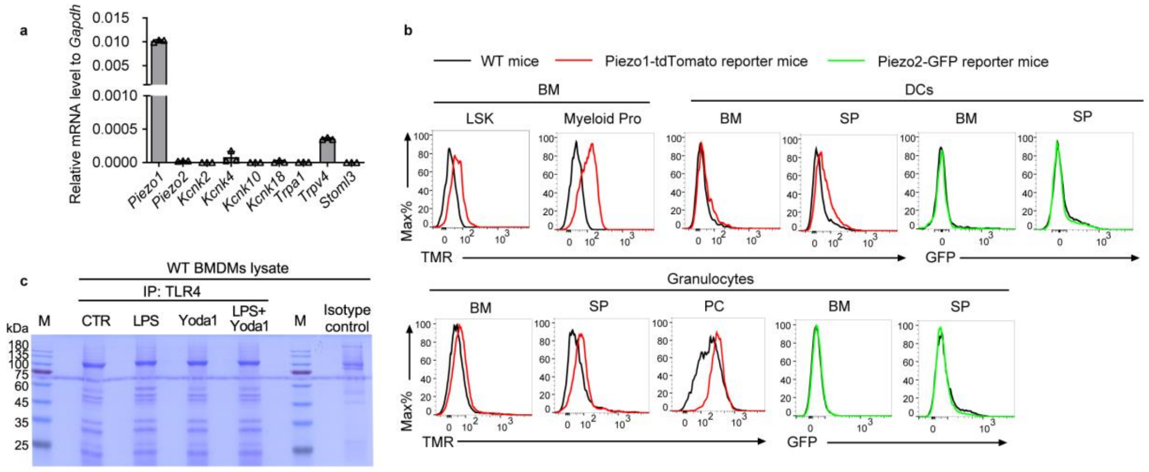

Supplementary Figure 1. **Expression of mechanosensory ion channels in myeloid cells. a** RT–qPCR analysis of the mRNA levels of known mammalian mechanosensory ion channels from unstimulated BMDMs (n = 3 technical replicates). Data are presented as mean +/- SD. Source data are provided as a Source Data file. **b** Flow cytometry histograms of tdTomato red fluorescence (TMR) or GFP from LSK cells (Linage$^-$Sca-1$^+$c-kit$^+$), myeloid progenitor cells (Myeloid Pro, Linage$^-$Sca-1$^-$c-kit$^+$), dendritic cells (DC, CD11c$^+$), and granulocytes (CD11b$^+$Gr-1$^+$) from bone marrow (BM), spleen (SP) or peritoneal cavity (PC) in *Piezo1*$^{\text{P1-tdT}}$ mice or *Piezo2*-EGFP-IRES-Cre mice. **c** The PAGE gel for mass spectrometry in immunoprecipitation assays using TLR4 antibody in cell lysates of BMDMs untreated or treated with LPS (1 μg ml^{-1}), Yoda1 (5 μM) or both. IP, immunoprecipitation; M, molecular size markers. Data are representative of three independent experiments with similar results.

Supplementary Figure 2

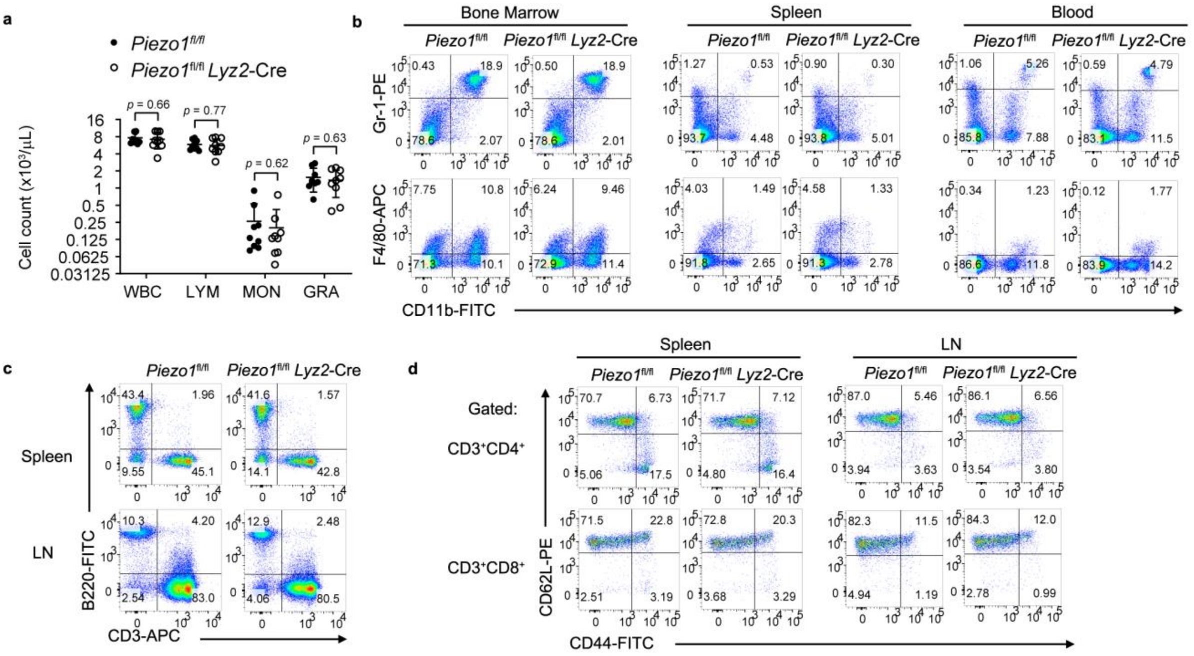

Supplementary Figure 2. **The myeloid cell specific Piezo1 knock out mice. a** White blood cell (WBC), circulating lymphocyte (LYM), monocyte (MON) and granulocyte (GRA) counts in *Piezo1*$^{fl/fl}$ and *Piezo1*$^{fl/fl}$ *Lyz2*-Cre mice. Each dot represents an individual mouse, n = 18 mice. Data are presented as mean +/- SD and the *p*-values of two-tailed unpaired Student's t test are indicated. Source data are provided as a Source Data file. **b** Flow cytometric analysis of Gr-1^{+}CD11b^{+} neutrophil and F4/80^{+}CD11b^{+} macrophage populations in the bone marrow, spleens or blood of *Piezo1*$^{fl/fl}$ and *Piezo1*$^{fl/fl}$ *Lyz2*-Cre mice, as determined with anti-Gr-1, anti-F4/80 and anti-CD11b antibodies. **c**, **d** Flow cytometric analysis of B220^{+} B cell and CD3^{+} T cell (**c**), the naïve T cell (CD62L^{high}CD44^{low}) and the effector T cell (CD62L^{low}CD44^{high}) (**d**) populations in the spleen or lymph node (LN) of *Piezo1*$^{fl/fl}$ and *Piezo1*$^{fl/fl}$ *Lyz2*-Cre mice, with the indicated antibodies. Data are representative of three independent experiments with similar results.

Supplementary Figure 3

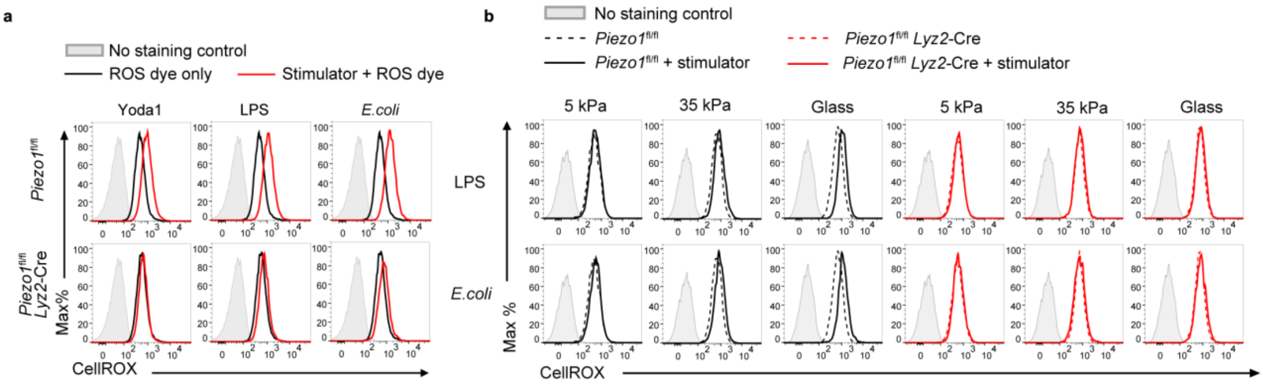

Supplementary Figure 3. **Piezo1 is critical for ROS production in BMDMs. a** Flow cytometry analyzing cellular ROS production by *Piezo1*$^{fl/fl}$ or *Piezo1*$^{fl/fl}$ *Lyz2*-Cre BMDMs stimulated with Yoda1 (5 μM) for 30 min, LPS (1 μg ml^{-1}) for 3 h or *E.coli* (MOI, 20), followed by staining with CellROX. **b** Flow cytometry analyzing cellular ROS production by *Piezo1*$^{fl/fl}$ or *Piezo1*$^{fl/fl}$ *Lyz2*-Cre BMDMs treated LPS (1 μg ml^{-1}) for 3 h or *E.coli* (MOI, 20) for 1 h on different stiffness culture matrix of 5kPa, 35 kPa, or glass, followed by staining with CellROX. Data are representative of three independent experiments with similar results.

Supplementary Figure 4

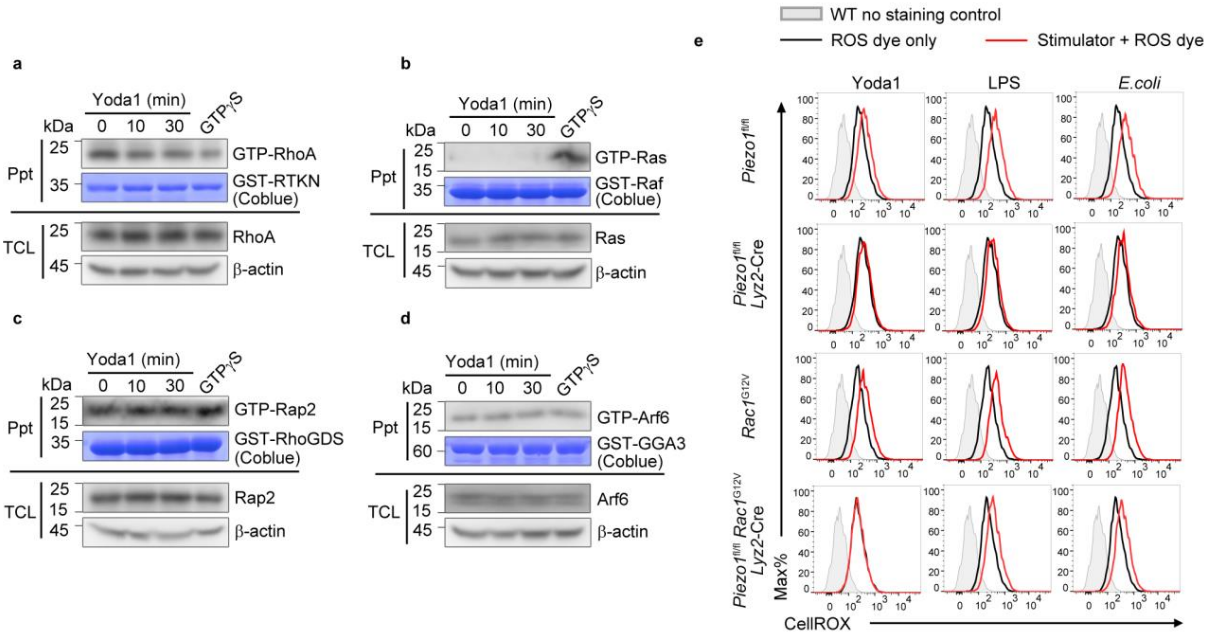

Supplementary Figure 4. **Piezo1 modulates cytoskeleton rearrangement via Rac1. a-d** Immunoblot analysis of the association of active (GTP-bound) RhoA with GST-RTKN^{RBD} (GST-RTKN) (**a**), GTP-Ras with GST-Raf^{RBD} (GTP-Raf) (**b**), GTP-Rap2 with GST-RhoGDS^{RBD} (GST-RhoGDS) (**c**) or GTP-Arf6 with GST-GGA^{RBD} (GTP-GGA) (**d**) in lysates of wild-type BMDMs treated with Yoda-1 (5 μM) for 0, 10 or 30 min. then incubated with the indicated GST-tagged detector; CoBlue, staining of GST-tagged detectors with Coomassie blue. Source data are provided as a Source Data file. **e** Flow cytometry analyzing cellular ROS production by *Piezo1*$^{fl/fl}$, *Piezo1*$^{fl/fl}$ *Lyz2*-Cre, *Rac1*^{G12V} *Lyz2*-Cre or *Piezo1*$^{fl/fl}$ *Rac1*^{G12V} *Lyz2*-Cre BMDMs stimulated with Yoda1 (5 μM) for 30 min, LPS (1 μg ml^{-1}) for 3 h or *E.coli* (MOI, 20) for 1 h, followed by staining with CellRox. Data are representative of three independent experiments with similar results.

Supplementary Figure 5

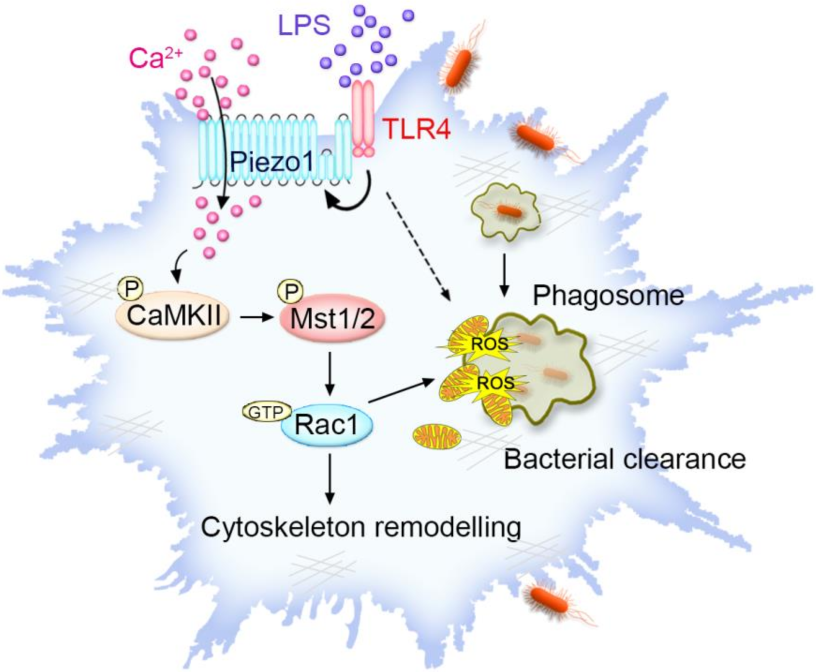

Supplementary Figure 5. **A working model of TLR4 coordinates Piezo1 to augment pathogen ingestion and killing**. TLR4 drives innate response partially through Piezo1 to promote F-actin reorganization and mitochondrion-phagosome juxtaposition for cytoskeleton remodelling, ROS generation and bacterial clearance.

Supplementary Figure 6

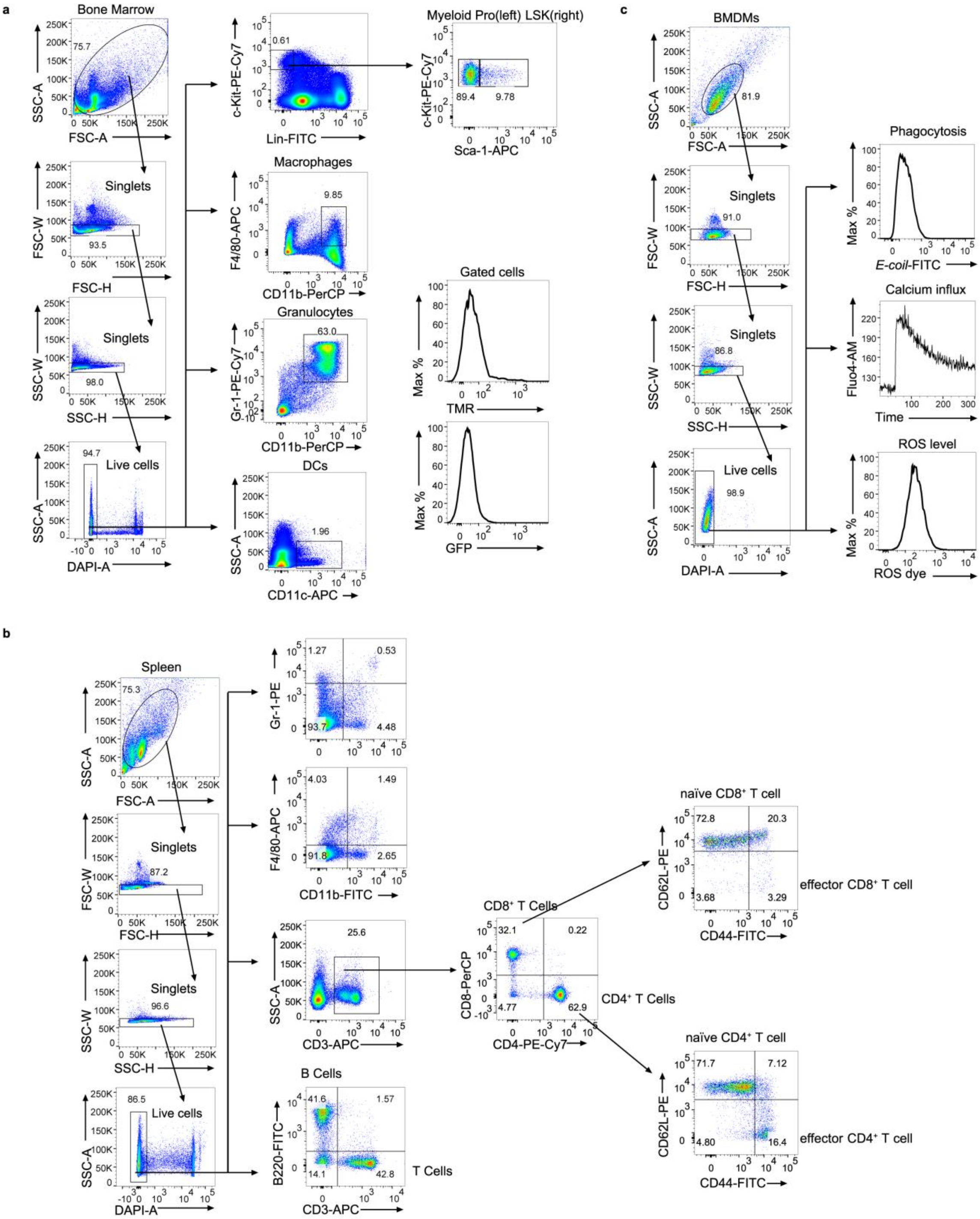

Supplementary Figure 6. **Flow cytometry gating strategy for Piezo1 expression, phagocytosis, calcium influx and ROS measurements. a** Representative flow cytometry dot plots and the employed gating strategy for measuring Piezo1 expression in indicated cells presented on Fig. 1a and Supplementary Figure 1b. **b** Representative flow cytometry dot plots and the employed gating strategy for measuring the immune cell composition of *Piezo1*$^{fl/fl}$ or *Piezo1*$^{fl/fl}$ *Lyz2*-Cre mice presented on Supplementary Figures 2b-d. **c** Representative flow cytometry dot plots and the employed gating strategy for *E. coli*-FITC phagocytosis, calcium influx and ROS analysis in BMDMs presented on Fig. 2e, 3g, 3h, 3j, 4c, 4i, 5h, 6a, 6k, Supplementary Figures 3a, 3b, 4e.

Supplementary Table 1. The sequence of primers used in the study

**qPCR primers**	**Forward primer sequence(5'-3')**	**Reverse primer sequence(5'-3')**
m. *Piezo1*	AGGACTTCCCCACCTATTGG	CCAGGGATGAGGATACTGGAAAA
m. *Piezo2*	GTGGTATGCAACCCAGTACCC	GGCCATTCTCTATGGGCAGG
m. *Kcnk2*	CCGAGGCTCTCATTCTCCTCA	AGGACGACCACCAGGAAAATC
m. *Kcnk4*	ATCTGGGGCTCTAGTGTTCCA	CCAAGCTGATGAGTGGTTGCT
m. *Kcnk10*	TGGCTGCATCGTGTTTGTGA	CTGTGGTCAGCGTGACTACC
m. *Kcnk18*	CTCTCTTCTCCGCTGTCGAG	AAGAGAGCGCTCAGGAAGG
m. *Trpa1*	GTCCAGGGCGTTGTCTATCG	CGTGATGCAGAGGACAGAGAT
m. *Trpv4*	ATGGCAGATCCTGGTGATGG	GGAACTTCATACGCAGGTTTGG
m. *Stoml3*	GATTCACCGGAGAAACTGGAG	TCCATACTGAGATTGGGAAGGT
m. *Gapdh*	AGGTCGGTGTGAACGGATTTG	TGTAGACCATGTAGTTGAGGTCA
