# Supplementary material for: TLR4 signalling via Piezo1 engages and enhances the macrophage mediated host response during bacterial infection
Source: Nat Commun. 2021 Jun 10;12:3519. doi: 10.1038/s41467-021-23683-y (PMC8192512; doi:10.1038/s41467-021-23683-y)
Supplement: Supplementary file 3 — Reporting Summary [file 41467_2021_23683_MOESM3_ESM.pdf]

nature research

Corresponding author(s): Dawang Zhou

Last updated by author(s): Apr 15, 2021

Reporting Summary

Nature Research wishes to improve the reproducibility of the work that we publish. This form provides structure for consistency and transparency in reporting. For further information on Nature Research policies, see our Editorial Policies and the Editorial Policy Checklist.

Statistics

For all statistical analyses, confirm that the following items are present in the figure legend, table legend, main text, or Methods section.

n/a	Confirmed	
☐	☒	The exact sample size (n) for each experimental group/condition, given as a discrete number and unit of measurement
☐	☒	A statement on whether measurements were taken from distinct samples or whether the same sample was measured repeatedly
☐	☒	The statistical test(s) used AND whether they are one- or two-sided<br>*Only common tests should be described solely by name; describe more complex techniques in the Methods section.*
☒	☐	A description of all covariates tested
☒	☐	A description of any assumptions or corrections, such as tests of normality and adjustment for multiple comparisons
☐	☒	A full description of the statistical parameters including central tendency (e.g. means) or other basic estimates (e.g. regression coefficient) AND variation (e.g. standard deviation) or associated estimates of uncertainty (e.g. confidence intervals)
☐	☒	For null hypothesis testing, the test statistic (e.g. F, t, r) with confidence intervals, effect sizes, degrees of freedom and P value noted<br>*Give P values as exact values whenever suitable.*
☒	☐	For Bayesian analysis, information on the choice of priors and Markov chain Monte Carlo settings
☒	☐	For hierarchical and complex designs, identification of the appropriate level for tests and full reporting of outcomes
☐	☒	Estimates of effect sizes (e.g. Cohen's d, Pearson's r), indicating how they were calculated

Our web collection on statistics for biologists contains articles on many of the points above.

Software and code

Policy information about availability of computer code

Data collection	AFM data was collected using NanoScope 9.40
Data analysis	AFM data was analyzed using NanoScope Analysis 1.80<br>Prism6 GraphPad www.graphpad.com/scientificsoftware/pri-sm<br>FlowJo v10 https://www.flowjo.com/solutions/flowjo/downloads<br>ImageJ 1.8.0<br>https://imagej.nih.gov/ij<br>Image-Pro Plus 6.0<br>https://image-pro-plus.software.informer.com/6.0/<br>BDFACSDiva.8.0<br>https://www.bdbiosciences.com/en-us/instruments/research-instruments/research-software/flow-cytometry-acquisition/facsdiva-software

For manuscripts utilizing custom algorithms or software that are central to the research but not yet described in published literature, software must be made available to editors and reviewers. We strongly encourage code deposition in a community repository (e.g. GitHub). See the Nature Research guidelines for submitting code & software for further information.

Data

Policy information about availability of data

All manuscripts must include a data availability statement. This statement should provide the following information, where applicable:

- Accession codes, unique identifiers, or web links for publicly available datasets
- A list of figures that have associated raw data
- A description of any restrictions on data availability

The data that support the findings of this study are available from the corresponding author upon reasonable request. Source data are provided with this paper. All unique materials used are readily available from the authors or from the standard commercial sources.

Field-specific reporting

Please select the one below that is the best fit for your research. If you are not sure, read the appropriate sections before making your selection.

☒ Life sciences ☐ Behavioural & social sciences ☐ Ecological, evolutionary & environmental sciences

For a reference copy of the document with all sections, see nature.com/documents/nr-reporting-summary-flat.pdf

Life sciences study design

All studies must disclose on these points even when the disclosure is negative.

Sample size	Sample size was determined accordingly to previous published study and experimental knowledge. ”Ref Kinases Mst1 and Mst2 positively regulate phagocytic induction of reactive oxygen species and bactericidal activity. Nat Immunol. 2015; Macrophage achieves self-protection against oxidative stress-induced ageing through the Mst-Nrf2 axis. Nat Commun. 2019.”
Data exclusions	No data were excluded from the experiments reported.
Replication	Data are from one experiment representative of three independent experiments with similar results.
Randomization	For animal experiments, the age and gender matched animals were randomized into control and treated groups. For other experiments, samples were randomly assigned to different experimental groups.
Blinding	The investigators were blinded to allocation during experiments and outcome assessment.

Behavioural & social sciences study design

All studies must disclose on these points even when the disclosure is negative.

Study description	*Briefly describe the study type including whether data are quantitative, qualitative, or mixed-methods (e.g. qualitative cross-sectional, quantitative experimental, mixed-methods case study).*
Research sample	*State the research sample (e.g. Harvard university undergraduates, villagers in rural India) and provide relevant demographic information (e.g. age, sex) and indicate whether the sample is representative. Provide a rationale for the study sample chosen. For studies involving existing datasets, please describe the dataset and source.*
Sampling strategy	*Describe the sampling procedure (e.g. random, snowball, stratified, convenience). Describe the statistical methods that were used to predetermine sample size OR if no sample-size calculation was performed, describe how sample sizes were chosen and provide a rationale for why these sample sizes are sufficient. For qualitative data, please indicate whether data saturation was considered, and what criteria were used to decide that no further sampling was needed.*
Data collection	*Provide details about the data collection procedure, including the instruments or devices used to record the data (e.g. pen and paper, computer, eye tracker, video or audio equipment) whether anyone was present besides the participant(s) and the researcher, and whether the researcher was blind to experimental condition and/or the study hypothesis during data collection.*
Timing	*Indicate the start and stop dates of data collection. If there is a gap between collection periods, state the dates for each sample cohort.*
Data exclusions	*If no data were excluded from the analyses, state so OR if data were excluded, provide the exact number of exclusions and the rationale behind them, indicating whether exclusion criteria were pre-established.*
Non-participation	*State how many participants dropped out/declined participation and the reason(s) given OR provide response rate OR state that no participants dropped out/declined participation.*
Randomization	*If participants were not allocated into experimental groups, state so OR describe how participants were allocated to groups, and if allocation was not random, describe how covariates were controlled.*

Ecological, evolutionary & environmental sciences study design

All studies must disclose on these points even when the disclosure is negative.

Study description	*Briefly describe the study. For quantitative data include treatment factors and interactions, design structure (e.g. factorial, nested, hierarchical), nature and number of experimental units and replicates.*
Research sample	*Describe the research sample (e.g. a group of tagged Passer domesticus, all Stenocereus thurberi within Organ Pipe Cactus National Monument), and provide a rationale for the sample choice. When relevant, describe the organism taxa, source, sex, age range and any manipulations. State what population the sample is meant to represent when applicable. For studies involving existing datasets, describe the data and its source.*
Sampling strategy	*Note the sampling procedure. Describe the statistical methods that were used to predetermine sample size OR if no sample-size calculation was performed, describe how sample sizes were chosen and provide a rationale for why these sample sizes are sufficient.*
Data collection	*Describe the data collection procedure, including who recorded the data and how.*
Timing and spatial scale	*Indicate the start and stop dates of data collection, noting the frequency and periodicity of sampling and providing a rationale for these choices. If there is a gap between collection periods, state the dates for each sample cohort. Specify the spatial scale from which the data are taken*
Data exclusions	*If no data were excluded from the analyses, state so OR if data were excluded, describe the exclusions and the rationale behind them, indicating whether exclusion criteria were pre-established.*
Reproducibility	*Describe the measures taken to verify the reproducibility of experimental findings. For each experiment, note whether any attempts to repeat the experiment failed OR state that all attempts to repeat the experiment were successful.*
Randomization	*Describe how samples/organisms/participants were allocated into groups. If allocation was not random, describe how covariates were controlled. If this is not relevant to your study, explain why.*
Blinding	*Describe the extent of blinding used during data acquisition and analysis. If blinding was not possible, describe why OR explain why blinding was not relevant to your study.*

Did the study involve field work? ☐ Yes ☐ No

Field work, collection and transport

Field conditions	*Describe the study conditions for field work, providing relevant parameters (e.g. temperature, rainfall).*
Location	*State the location of the sampling or experiment, providing relevant parameters (e.g. latitude and longitude, elevation, water depth).*
Access & import/export	*Describe the efforts you have made to access habitats and to collect and import/export your samples in a responsible manner and in compliance with local, national and international laws, noting any permits that were obtained (give the name of the issuing authority, the date of issue, and any identifying information).*
Disturbance	*Describe any disturbance caused by the study and how it was minimized.*

Reporting for specific materials, systems and methods

We require information from authors about some types of materials, experimental systems and methods used in many studies. Here, indicate whether each material, system or method listed is relevant to your study. If you are not sure if a list item applies to your research, read the appropriate section before selecting a response.

Materials & experimental systems

n/a	Involved in the study	
☐	☒	Antibodies
☐	☒	Eukaryotic cell lines
☒	☐	Palaeontology and archaeology
☐	☒	Animals and other organisms
☒	☐	Human research participants
☒	☐	Clinical data
☒	☐	Dual use research of concern

Methods

n/a	Involved in the study	
☒	☐	ChIP-seq
☐	☒	Flow cytometry
☒	☐	MRI-based neuroimaging

Antibodies

Antibodies used

All antibodies used in this study, including antibody specificity, supplier name and clone name are provided in the Methods section, the legends of the figures and in the supplementary files.
TLR4 Rabbit pAb, Proteintech, cat# 19811-1-AP, lot: 00048459.
RFP Rabbit pAb, Abcam, cat#ab62341, lot: GR3231879-3.
Hsp60 Rabbit mAb, Cell Signaling Technology, cat#4870, clone: D307, lot: 2.
Phospho-Mob1 (Thr35) Rabbit mAb, Cell Signaling Technology, cat#8699, D2F10, lot: 2.
Mob1 Rabbit mAb, Cell Signaling Technology, cat#13730, clone: E1N9D, lot: 2.
Mst1 Rabbit pAb, Cell Signaling Technology, cat#3682, lot: 5.
Mst2 Rabbit pAb, Cell Signaling Technology, cat#3952, lot: 7.
gamma-tubulin Mouse mAb, Abcam, cat#ab11316, clone: GTU-88, lot: GR3352565-1.
Phospho-P38 Rabbit pAb, Cell Signaling Technology, cat#9211, lot: 22.
Phospho-Jnk Rabbit mAb, Cell Signaling Technology, cat#4668, clone: 81E11, lot: 7.
Rac1 Rabbit mAb, Proteintech, cat#66122-1-lg, clone: 4A4B11, lot: 10017770.
beta-Actin Rabbit mAb, Cell Signaling Technology, cat#8457, clone: D6A8, lot: 7.
GAPDH Rabbit mAb, Cell Signaling Technology, cat#5174, clone: D16H11, lot: 8.
HA tag Rabbit pAb, Proteintech, cat#51064-2-AP, lot: 00082685.
DYKDDDDK (Flag) Rabbit pAb, Proteintech, cat# 20543-1-AP, lot: 00056378.
pan Ras Mouse mAb, Proteintech, cat# 60309-1-Ig, clone: 4H4G7, lot: 10004103.
ARF6 Rabbit pAb, Proteintech, cat# 20225-1-AP, lot: 00041861.
RAP2 Rabbit pAb, Proteintech, cat# 13789-1-AP, lot: 00057533.
RhoA Rabbit mAb, Cell Signaling Technology, cat#2117, clone: 67B9, lot: 2.
Cdc42 Rabbit mAb, Cell Signaling Technology, cat#2466, clone: 11A11, lot: 4.
Alexa Fluor 488 Donkey anti-Mouse IgG, Invitrogen, cat#A21202, lot: 2090565.
Alexa Fluor 555 Donkey anti-Rabbit IgG, Invitrogen, cat#A31572, lot: 2088692.
PerCP/Cyanine5.5 anti-mouse/human CD11b Antibody, Biolegend, cat#101228, clone: M1/70, lot: B308468.
APC anti-mouse F4/80 Antibody, Biolegend, cat#123116, clone: BM8, lot: B268075.
PE Rat Anti-Mouse CD62L Antibody, BD, cat# 553151, clone: MEL-14, lot: 5181587.
FITC anti-mouse/human CD44 Antibody, Biolegend, cat# 103006, clone: IM7, lot: B228504.
APC anti-mouse CD3ε Antibody, Biolegend, cat# 100312, clone: 145-2C11, lot: B304829.
FITC anti-mouse/human CD45R/B220 Antibody, Biolegend, cat# 103206, clone: RA3-6B2, lot: B209103.
PE anti-mouse Ly-6G/Ly-6C (Gr-1) Antibody, BD, cat#553128, clone: RB6-8C5, lot: 9098937.
APC anti-mouse CD11c Antibody, Biolegend, cat#117310, clone: N418, lot: B233903.
APC anti-mouse Ly-6A/E (Sca-1) Antibody, Biolegend, cat#108112, clone: D7, lot: B265429.
PE/Cy7 anti-mouse Ly-6G/Ly-6C (Gr-1) Antibody, Biolegend, cat#108416, clone: RB6-8C5, lot: B284962.
PE/Cy7 anti-mouse CD117 (c-Kit) Antibody, Biolegend, cat#105814, clone: 2B8, lot:B252918.
FITC anti-mouse Lineage Cocktail, Biolegend, cat#133302, clone: 145-2C11; RB6-8C5; RA3-6B2; Ter-119; M1/70, lot: B281150.
FITC anti-mouse/human CD11b Antibody, Biolegend, cat#101206, clone: M1/70, lot: B224362.

Validation

All antibodies were obtained commercially were tested and validated by the respective company. All antibodies had validation statement provided on the website of the manufacturer.
TLR4 Rabbit pAb: Immunofluorescence analysis of murine macrophages, staining TLR4 antibody at dilution of 1:500. (see manufacturer's website (https://www.ptglab.com/Products/Pictures/pdf/19811-1-AP.pdf) or Xu A et al. Overexpressed P75CUX1 promotes EMT in glioma infiltration by activating β-catenin. Cell Death Dis. 2021)
RFP Rabbit pAb: Immunofluorescent analysis of Mouse Epiblast stem cells using RFP antibody at dilution of 1: 100. (see manufacturer's website (https://www.abcam.com/rfp-antibody-ab62341.pdf) or Xue M et al. Equalizing excitation-inhibition ratios across visual cortical neurons. Nature. 2014)
Hsp60 Rabbit mAb: Immunofluorescent analysis of HeLa cells using HSP60 at dilution of 1:100. (see manufacturer's website: https://www.cellsignal.com/datasheet.jsp?productId=4870&images=1)
Phospho-Mob1 (Thr35) Rabbit mAb: Western blot analysis of extracts from MCF7 cells using Phospho-MOB1 Rabbit mAb at dilution of 1:1000. (see manufacturer's website: https://www.cellsignal.com/datasheet.jsp?productId=8699&images=1)
Mob1 Rabbit mAb: Western blot analysis of of extracts from various cell lines using MOB1 Rabbit mAb at dilution of 1:1000. (see manufacturer's website: https://www.cellsignal.com/datasheet.jsp?productId=13730&images=1)
Mst1 Rabbit pAb: Western blot analysis of extracts from various cell lines using MST1 Antibody at dilution of 1:1000. (see manufacturer's website: https://www.cellsignal.com/datasheet.jsp?productId=3682&images=1)
Mst2 Rabbit pAb: Western blot analysis of extracts from various cell lines using MST2 Antibody at dilution of 1:1000. (see manufacturer's website: https://www.cellsignal.com/datasheet.jsp?productId=3952&images=1)
gamma-tubulin Mouse mAb: Western blot analysis of of extracts from various cell lines using gamma-tubulin Rabbit mAb at dilution of 1:10000. (see manufacturer's website: https://www.abcam.com/gamma-tubulin-antibody-gtu-88-centrosome-marker-ab11316.pdf)
Phospho-P38 Rabbit pAb: Western blot analysis of extracts from C6 cells using Phospho-p38 MAPK (Thr180/Tyr182) Antibody at dilution of 1:1000. (see manufacturer's website: https://www.cellsignal.com/datasheet.jsp?productId=9211&images=1)
Phospho-Jnk Rabbit mAb: Western blot analysis of extracts from 293 cells using Phospho-SAPK/JNK Rabbit mAb at dilution of 1:1000. (see manufacturer's website: https://www.cellsignal.com/datasheet.jsp?productId=4668&images=1)
Rac1 Rabbit mAb: Western blot analysis of extracts from NIH3T3 cells using Rac1 Antibody at dilution of 1:1000. (see manufacturer's website: https://www.ptglab.com/Products/Pictures/pdf/66122-1-Ig.pdf)

beta-Actin Rabbit mAb: Western blot analysis of extracts from various cell lines using beta-Actin Rabbit mAb at dilution of 1:1000. (see manufacturer's website: https://www.cellsignal.com/datasheet.jsp?productId=8457&images=1)
GAPDH Rabbit mAb: Western blot analysis of extracts from various cell lines using GAPDH Rabbit mAb at dilution of 1:1000. (see manufacturer's website: https://www.cellsignal.com/datasheet.jsp?productId=5147&images=1)
HA tag Rabbit pAb: Western blot analysis of HA-tagged fusion protein with anti-HA-tag Rabbit mAb at various dilutions. (see manufacturer's website: https://www.ptglab.com/Products/Pictures/pdf/51064-2-AP.pdf)
DYKDDDDK (Flag) Rabbit pAb: Western blot analysis of DYKDDDDK-tagged fusion protein with anti-HA-tag Rabbit mAb at various dilutions. (see manufacturer's website: https://www.ptglab.com/Products/Pictures/pdf/20543-1-AP.pdf)
pan Ras Mouse mAb: Western blot analysis of extracts from various cell lines using beta-Actin Rabbit mAb at dilution of 1:1000. (see manufacturer's website: https://www.ptglab.com/Products/Pictures/pdf/60309-1-Ig.pdf)
ARF6 Rabbit pAb: Western blot analysis of extracts from HepG2 cells using ARF6 Antibody at dilution of 1:1000. (see manufacturer's website: https://www.ptglab.com/Products/Pictures/pdf/20225-1-AP.pdf)
RAP2 Rabbit pAb: Western blot analysis of extracts from A431 cells using RAP2 Antibody at dilution of 1:1000. (see manufacturer's website: https://www.ptglab.com/Products/Pictures/pdf/13789-1-AP.pdf)
RhoA Rabbit mAb: Western blot analysis of extracts from various cell lines using RhoA Rabbit mAb at dilution of 1:1000. (see manufacturer's website: https://www.cellsignal.com/datasheet.jsp?productId=2117&images=1)
Cdc42 Rabbit mAb: Western blot analysis of extracts from various cell lines using Cdc42 Rabbit mAb at dilution of 1:1000. (see manufacturer's website: https://www.cellsignal.com/datasheet.jsp?productId=2462&images=1)
Alexa Fluor 488 Donkey anti-Mouse IgG: Immunofluorescence analysis of Donkey anti-Mouse IgG (H+L) Secondary Antibody, Alexa Fluor 488 conjugate (A21202, at dilution of 1:300) was performed using MCF-7 cells stained with Cytokeratin 19 Mouse Monoclonal Antibody. (see manufacturer's website: https://www.thermofisher.com/order/genome-database/dataSheetPdf?producttype=antibody&productsubtype=antibody_secondary&productId=A-21202&version=137)
Alexa Fluor 555 Donkey anti-Rabbit IgG: Immunofluorescence analysis of Donkey anti-Rabbit IgG (H+L) Highly Cross-Adsorbed Secondary Antibody Alexa Fluor® 555 (A31572, at dilution of 1:300) conjugate was performed using HeLa cells stained with alpha Tubulin Rabbit Polyclonal Antibody. (see manufacturer's website: https://www.thermofisher.com/order/genome-database/dataSheetPdf?producttype=antibody&productsubtype=antibody_secondary&productId=A-31572&version=137)
PerCP/Cyanine5.5 anti-mouse/human CD11b Antibody: C57BL/6 splenocytes were blocked with TruStainFcX™ (anti-mouse CD16/32) Antibody then stained with CD11b (clone M1/70) PerCP/Cyanine5.5. (see manufacturer's website: https://www.biolegend.com/en-us/products/percp-cyanine5-5-anti-mouse-human-cd11b-antibody-4257)

APC anti-mouse F4/80 Antibody: Thioglycolate-elicited BALB/c mouse peritoneal macrophages stained with F4/80 (clone BM8) APC. (see manufacturer's website: https://www.biolegend.com/en-us/products/apc-anti-mouse-f4-80-antibody-4071)
PE Rat Anti-Mouse CD62L Antibody: C57BL/6 mouse bone marrow cells were stained with CD62L (clone MEL-14) PE. (see manufacturer's website: https://www.bdbiosciences.com/ds/pm/tds/553151.pdf)
FITC anti-mouse/human CD44 Antibody: C57BL/6 mouse splenocytes stained with CD44 (clone IM7) FITC. (see manufacturer's website: https://www.biolegend.com/en-us/products/fitc-anti-mouse-human-cd44-antibody-314?GroupID=GROUP20)
APC anti-mouse CD3ε Antibody: C57BL/6 mouse splenocytes were stained with CD3e (clone 145-2C11) APC. (see manufacturer's website: https://www.biolegend.com/en-us/products/apc-anti-mouse-cd3epsilon-antibody-21)
FITC anti-mouse/human CD45R/B220 Antibody: C57BL/6 mouse splenocytes stained with CD45R/B220 (clone RA3-6B2) FITC. (see manufacturer's website: https://www.biolegend.com/en-us/products/fitc-anti-mouse-human-cd45r-b220-antibody-445)
PE anti-mouse Ly-6G/Ly-6C (Gr-1) Antibody: C57BL/6 mouse bone marrow cells were stained with Ly-6G/Ly-6C (clone RB6-8C5) PE. (see manufacturer's website: https://www.bdbiosciences.com/ds/pm/tds/553128.pdf
APC anti-mouse CD11c Antibody: C57BL/6 mouse splenocytes stained with CD11c (clone N418) APC. (see manufacturer's website: https://www.biolegend.com/en-us/products/apc-anti-mouse-cd11c-antibody-1813)
APC anti-mouse Ly-6A/E (Sca-1) Antibody: C57BL/6 mouse splenocytes stained with Ly-6A/E (Sca-1) (clone D7) APC. (see manufacturer's website: https://www.biolegend.com/en-us/products/apc-anti-mouse-ly-6a-e-sca-1-antibody-225)
PE/Cy7 anti-mouse Ly-6G/Ly-6C (Gr-1) Antibody: C57BL/6 mouse bone marrow cells were stained with Ly-6G/Ly-6C (clone RB6-8C5) PE/Cy7. (see manufacturer's website: https://www.biolegend.com/en-us/products/pe-cyanine7-anti-mouse-ly-6g-ly-6c-gr-1-antibody-1931)
PE/Cy7 anti-mouse CD117 (c-Kit) Antibody: C57BL/6 bone marrow cells stained with CD117 (c-Kit) (clone 2B8) PE/Cyanine7. (see manufacturer's website: https://www.biolegend.com/en-us/products/pe-cyanine7-anti-mouse-cd117-c-kit-antibody-1900)
FITC anti-mouse Lineage Cocktail: C57BL/6 bone marrow cells stained with FITC Lineage Cocktail. (see manufacturer's website: https://www.biolegend.com/en-us/products/fitc-anti-mouse-lineage-cocktail-with-isotype-ctrl-5803)
FITC anti-mouse/human CD11b Antibody: C57BL/6 mouse bone marrow cells were stained with CD11b (clone M1/70) FITC. (see manufacturer's website: https://www.biolegend.com/en-us/products/fitc-anti-mouse-human-cd11b-antibody-347)

Eukaryotic cell lines

Policy information about cell lines

Cell line source(s)	BMDMs were derived from mouse bone marrow cells with recombinant mouse M-CSF.<br>The 293T cell line was originally obtained from the American Type Culture Collection.
Authentication	293T cells were purchased from ATCC, and the authentication was also provided by ATCC
Mycoplasma contamination	All cells were tested for mycoplasma contamination and were found to be negative.
Commonly misidentified lines<br>(See ICLAC register)	No commonly misidentified cell lines were used.

Palaeontology and Archaeology

Specimen provenance	*Provide provenance information for specimens and describe permits that were obtained for the work (including the name of the issuing authority, the date of issue, and any identifying information).*
Specimen deposition	*Indicate where the specimens have been deposited to permit free access by other researchers.*
Dating methods	*If new dates are provided, describe how they were obtained (e.g. collection, storage, sample pretreatment and measurement), where they were obtained (i.e. lab name), the calibration program and the protocol for quality assurance OR state that no new dates are provided.*

☐ Tick this box to confirm that the raw and calibrated dates are available in the paper or in Supplementary Information.

Ethics oversight	*Identify the organization(s) that approved or provided guidance on the study protocol, OR state that no ethical approval or guidance was required and explain why not.*

Note that full information on the approval of the study protocol must also be provided in the manuscript.

Animals and other organisms

Policy information about studies involving animals; ARRIVE guidelines recommended for reporting animal research

Laboratory animals	Male mice between 6-8 weeks of age were used. Wild-type C57BL/6 mice, Piezo1P1-tdT mice (029214) ,Piezo1flox mice (029213), Piezo2-EGFP-IRES-Cre mice (027719), Rac1flox mice (005550), C57BL/6-Gt(ROSA)26Sortm9(Rac1*,EGFP)Rsky/J mice (012361) C57BL/10ScNJ (Tlr4lps-del, 003752) and Lyz2-Cre mice (004781) were originally from the Jackson Laboratory. Mst1fl/fl mice were from Dr. R. L. Johnson's lab, University of Texas, M.D. Anderson Cancer Center, Houston, USA Mst2fl/fl mouse was described in Zhou D, et al, Cancer Cell. 2009 Nov 6;16(5):425-38.
Wild animals	The study does not involve wild animals.
Field-collected samples	The study does not involve field-collected samples.
Ethics oversight	All Mice were housed under specific pathogen-free conditions with a 12h light/dark cycle, at a temperature of 22 ± 2 °C and a relative humidity of 50 ± 5%, and were fed with a standard mouse chow diet at the Xiamen University Laboratory Animal Center. These mouse experiments were approved by the Institutional Animal Care and Use Committee and were in strict accordance with good animal practice as defined by the Xiamen University Laboratory Animal Center.

Note that full information on the approval of the study protocol must also be provided in the manuscript.

Human research participants

Policy information about studies involving human research participants

Population characteristics	*Describe the covariate-relevant population characteristics of the human research participants (e.g. age, gender, genotypic information, past and current diagnosis and treatment categories). If you filled out the behavioural & social sciences study design questions and have nothing to add here, write "See above."*
Recruitment	*Describe how participants were recruited. Outline any potential self-selection bias or other biases that may be present and how these are likely to impact results.*
Ethics oversight	*Identify the organization(s) that approved the study protocol.*

Note that full information on the approval of the study protocol must also be provided in the manuscript.

Clinical data

Policy information about clinical studies

All manuscripts should comply with the ICMJEguidelines for publication of clinical research and a completedCONSORT checklist must be included with all submissions.

Clinical trial registration	*Provide the trial registration number from ClinicalTrials.gov or an equivalent agency.*
Study protocol	*Note where the full trial protocol can be accessed OR if not available, explain why.*
Data collection	*Describe the settings and locales of data collection, noting the time periods of recruitment and data collection.*
Outcomes	*Describe how you pre-defined primary and secondary outcome measures and how you assessed these measures.*

Dual use research of concern

Policy information about dual use research of concern

Hazards

Could the accidental, deliberate or reckless misuse of agents or technologies generated in the work, or the application of information presented in the manuscript, pose a threat to:

No	Yes	
☐	☐	Public health
☐	☐	National security
☐	☐	Crops and/or livestock
☐	☐	Ecosystems
☐	☐	Any other significant area

Experiments of concern

Does the work involve any of these experiments of concern:

No	Yes	
☐	☐	Demonstrate how to render a vaccine ineffective
☐	☐	Confer resistance to therapeutically useful antibiotics or antiviral agents
☐	☐	Enhance the virulence of a pathogen or render a nonpathogen virulent
☐	☐	Increase transmissibility of a pathogen
☐	☐	Alter the host range of a pathogen
☐	☐	Enable evasion of diagnostic/detection modalities
☐	☐	Enable the weaponization of a biological agent or toxin
☐	☐	Any other potentially harmful combination of experiments and agents

ChIP-seq

Data deposition

☐ Confirm that both raw and final processed data have been deposited in a public database such as GEO.

☐ Confirm that you have deposited or provided access to graph files (e.g. BED files) for the called peaks.

Data access links<br>*May remain private before publication.*	*For "Initial submission" or "Revised version" documents, provide reviewer access links. For your "Final submission" document, provide a link to the deposited data.*
Files in database submission	*Provide a list of all files available in the database submission.*
Genome browser session<br>(e.g. UCSC)	*Provide a link to an anonymized genome browser session for "Initial submission" and "Revised version" documents only, to enable peer review. Write "no longer applicable" for "Final submission" documents.*

Methodology

Replicates	*Describe the experimental replicates, specifying number, type and replicate agreement.*
Sequencing depth	*Describe the sequencing depth for each experiment, providing the total number of reads, uniquely mapped reads, length of reads and whether they were paired- or single-end.*
Antibodies	*Describe the antibodies used for the ChIP-seq experiments; as applicable, provide supplier name, catalog number, clone name, and lot number.*
Peak calling parameters	*Specify the command line program and parameters used for read mapping and peak calling, including the ChIP, control and index files used.*
Data quality	*Describe the methods used to ensure data quality in full detail, including how many peaks are at FDR 5% and above 5-fold enrichment.*
Software	*Describe the software used to collect and analyze the ChIP-seq data. For custom code that has been deposited into a community repository, provide accession details.*

Flow Cytometry

Plots

Confirm that:

- [x] The axis labels state the marker and fluorochrome used (e.g. CD4-FITC).
- [x] The axis scales are clearly visible. Include numbers along axes only for bottom left plot of group (a 'group' is an analysis of identical markers).
- [x] All plots are contour plots with outliers or pseudocolor plots.
- [x] A numerical value for number of cells or percentage (with statistics) is provided.

Methodology

Sample preparation	Single cells isolated from the bone marrow, spleen or peritoneal cavity were stained for 30 min with the appropriate fluorescence-conjugated antibodies and washed, then were resuspended with flow cytometry staining buffer (2% FBS in PBS) containing DAPI (4′,6-diamidino-2-phenylindole; Invitrogen).<br>Cells (BMDMs) were plated in non–tissue-culture-treated dishes. Samples were treated with stimulants for indicated time as needed. The culture medium was removed and then the cells were washed with PBS and then incubated for 30 min at 37℃ with MitoSOX (for measurement of mROS superoxide; Invitrogen) and/or CellROX (for measurement of total cellular H2O2; Invitrogen) at a final concentration of 5 µM in serum-free DMEM (Invitrogen). The cells were washed with warmed PBS, removed from the plates by pipetting with 1% trypsin containing 1 mM EDTA, pelleted at 1,600 r.p.m. for 3 min, immediately re-suspended in cold PBS containing 1% FBS and analyzed by flow cytometry.
Instrument	BD LSRFortessa flow cytometer (BD Biosciences)
Software	Data were collected with BD FACSDIVA™ SOFTWARE and analyzed with FlowJo software (TreeStar)
Cell population abundance	More than 95%
Gating strategy	Titration of the antibodies was performed to determine the optimal concentration of the antibodies for staining of cells. FMO (Flourescence Minus One) assay were used to determine fluorochrome overlap and set negative gate.<br>Unstained cells were used to set gates. Single antibody stained cells were used to define positive gates.

- [x] Tick this box to confirm that a figure exemplifying the gating strategy is provided in the Supplementary Information.

Magnetic resonance imaging

Experimental design

Design type	*Indicate task or resting state; event-related or block design.*
Design specifications	*Specify the number of blocks, trials or experimental units per session and/or subject, and specify the length of each trial or block (if trials are blocked) and interval between trials.*
Behavioral performance measures	*State number and/or type of variables recorded (e.g. correct button press, response time) and what statistics were used to establish that the subjects were performing the task as expected (e.g. mean, range, and/or standard deviation across subjects).*

Acquisition

Imaging type(s)	*Specify: functional, structural, diffusion, perfusion.*
Field strength	*Specify in Tesla*
Sequence & imaging parameters	*Specify the pulse sequence type (gradient echo, spin echo, etc.), imaging type (EPI, spiral, etc.), field of view, matrix size, slice thickness, orientation and TE/TR/flip angle.*
Area of acquisition	*State whether a whole brain scan was used OR define the area of acquisition, describing how the region was determined.*

Diffusion MRI ☐ Used ☐ Not used

Preprocessing

Preprocessing software	*Provide detail on software version and revision number and on specific parameters (model/functions, brain extraction, segmentation, smoothing kernel size, etc.).*
Normalization	*If data were normalized/standardized, describe the approach(es): specify linear or non-linear and define image types used for transformation OR indicate that data were not normalized and explain rationale for lack of normalization.*

Normalization template

Describe the template used for normalization/transformation, specifying subject space or group standardized space (e.g. original Talairach, MNI305, ICBM152) OR indicate that the data were not normalized.

Noise and artifact removal

Describe your procedure(s) for artifact and structured noise removal, specifying motion parameters, tissue signals and physiological signals (heart rate, respiration).

Volume censoring

Define your software and/or method and criteria for volume censoring, and state the extent of such censoring.

Statistical modeling & inference

Model type and settings

Specify type (mass univariate, multivariate, RSA, predictive, etc.) and describe essential details of the model at the first and second levels (e.g. fixed, random or mixed effects; drift or auto-correlation).

Effect(s) tested

Define precise effect in terms of the task or stimulus conditions instead of psychological concepts and indicate whether ANOVA or factorial designs were used.

Specify type of analysis: ☐ Whole brain ☐ ROI-based ☐ Both

Statistic type for inference
(See Eklund et al. 2016)

Specify voxel-wise or cluster-wise and report all relevant parameters for cluster-wise methods.

Correction

Describe the type of correction and how it is obtained for multiple comparisons (e.g. FWE, FDR, permutation or Monte Carlo).

Models & analysis

n/a	Involved in the study
☐	☐ Functional and/or effective connectivity
☐	☐ Graph analysis
☐	☐ Multivariate modeling or predictive analysis

Functional and/or effective connectivity

Report the measures of dependence used and the model details (e.g. Pearson correlation, partial correlation, mutual information).

Graph analysis

Report the dependent variable and connectivity measure, specifying weighted graph or binarized graph, subject- or group-level, and the global and/or node summaries used (e.g. clustering coefficient, efficiency, etc.).

Multivariate modeling and predictive analysis

Specify independent variables, features extraction and dimension reduction, model, training and evaluation metrics.
